# Commentary – HIV-Induced Extracranial Carotid Ectasia and Stroke

**Published:** 2021

**Authors:** Rakhee Lalla, Prashant Raghavan, John W. Cole

**Affiliations:** 1 Department of Neurology, University of Maryland School of Medicine, Baltimore, MD, 21201, USA; 2 Department of Radiology, University of Maryland School of Medicine, Baltimore, MD, 21201, USA; 3 Departments of Neurology, Veterans Affairs Maryland Health Care System and the University of Maryland School of Medicine, Baltimore, MD, 21201, USA

HIV is a known risk factor for both ischemic and hemorrhagic stroke. Even with the widespread use of antiretroviral therapy, stroke incidence is higher in patients with HIV compared to non-HIV control subjects [1]. Ischemic stroke in patients with HIV are often deemed to be cryptogenic, but several possible etiologies for stroke have been identified in this population, including coagulopathy, opportunistic infection, cardioembolism and vasculopathy [2,3]. Our recent publication describes several cases of HIV vasculopathy with development of extracranial ectatic vasculature contributing to acute ischemic stroke in these patients [4]. We further described the various etiologies of stroke in the HIV population, emphasizing the pathophysiology of HIV-induced vasculopathy. In the present commentary, we briefly summarize the critical aspects of our recent publication and highlight our suggested algorithm for a more comprehensive work up of ischemic stroke in HIV positive patients.

HIV has been found to be associated with a variety of coagulopathies including Protein C and S deficiencies as well as antiphospholipid syndrome. The precise risk relationships between these coagulopathies in the setting of HIV and stroke has yet to be established. Opportunistic infections, specifically varicella zoster, tuberculosis and neurosyphilis have also been implicated with stroke in the HIV population. There is thought to be a higher incidence of cardioembolism in patients with HIV, due to an increased risk of atrial fibrillation and cardiomyopathy, as well as endocarditis leading to septic emboli [1]. Finally, HIV vasculopathy, in the form of atherosclerotic and non-atherosclerotic disease as well as vasculitis, are known etiologies of stroke in this patient population via varied mechanisms. The ischemic stroke cases described in our previous publication demonstrate extracranial non-atherosclerotic vasculopathy leading to vessel ectasia and subsequent thrombus formation [4].

Vasculopathy is known to be one of the major etiologies of stroke in the HIV positive population. Studies have worked to classify HIV vasculopathy into three subsets: 1) vasculitis, 2) accelerated atherosclerosis, and 3) non-atherosclerotic vasculopathy with intimal hyperplasia; although there is typically some overlap among these three mechanisms [3]. The pro-inflammatory state associated with HIV is thought to cause endothelial damage and intimal hyperplasia which contributes to accelerated atherosclerosis [1,2]. Non-atherosclerotic vasculopathy on the other hand, leads to vessel ectasia through vessel wall disruption and then subsequent thrombus formation. These changes can occur both extra- and intra-cranially. Several mechanisms for ectasia have been identified including fragmentation of the internal elastic lamina and thinning of the media layer [5–7]. The pathophysiology for these changes is not well defined, although is thought to be related to impairment in matrix metalloproteinases, which have also been associated with aneurysmal dilation in abdominal-aortic and coronary arteries [8–10].

The clinical presentation of stroke related to arterial ectasia (dilated) and dolichoectasia (dilated and elongated) can be variable. Some studies suggest manifestations of small vessel disease including lacunar infarcts and cerebral microbleeds [11]. As described in our cases, large vessel involvement can lead to thrombus formation in dilated extracranial vasculature leading to an embolic stroke presentation. Intracranially, dolichoectatic vasculature may be present in the posterior circulation and can cause mass effect on structures in the posterior fossa, including the brainstem. Thus, it is important to evaluate for, and to recognize the effects of, these variant vessel anatomies in the setting of HIV.

Critically, the work up and management of stroke in the HIV population must be tailored given the various possible etiologies. Although all patients must receive the standard “heart to head” stroke work up, it is important to include additional testing for alternative etiologies that are relevant to this patient population. As consistent with our prior publication [4], we propose an algorithm for evaluation of stroke in HIV positive patients as outlined in Figure 1. In addition to evaluation of the heart, proximal aorta and vasculature of the head and neck, it is important to assess HIV status by CD4 count and viral load to determine the risk for opportunistic infection. Further imaging should be pursued if there is a concern for vasculitis or vasculopathy related to HIV. Most studies suggest high resolution MRA with gadolinium enhancement as a means of evaluating HIV vasculopathy. If irregular ectatic vasculature is identified, clinicians should consider this as a potential embolic source, with serial imaging to monitor for progression over time [8].

There is limited data for secondary prevention in patients with HIV. Thus, much of our clinical decision making in preventive management is extrapolated from that of the general population, with use of an antiplatelet agents, anticoagulation when indicated, and high-intensity statin therapy. Statins are thought to have a dual benefit in patients with HIV, targeting both dyslipidemia and the pro-inflammatory state. Trials are underway to further define therapies that may diminish the inflammatory response in this particular population [2]. The long-term management of patients with vessel ectasia in this setting is also poorly defined but addressing and optimizing all vascular risk factors over both the near- and long-term is critical. The pathophysiology for ectasia/dolichoectasia is thought to share mechanisms between small and large vessel disease, and therefore aggressive management of all vascular risk factors is crucial in secondary prevention [11]. Literature suggests long-term treatment with anti-platelet agents as opposed to anticoagulation given the higher risk of intracranial hemorrhage in patients with vessel ectasia [8,9].

In addition to the stroke etiologies described above, more recent studies have commented on the role of antiretroviral therapy (ART) in stroke pathogenesis in the HIV population. Specifically, prolonged use of protease inhibitors has been shown to be associated with dyslipidemia and therefore increased risk of stroke and other cardiovascular disease [1,2]. Several classes of ART drugs have also demonstrated potential to increase stroke risk by inducing endothelial toxicity and vascular dysfunction in HIV [1]. HIV itself is thought to alter blood brain barrier permeability as well, and certain ARTs may contribute to this further, promoting movement of viral infected and inflammatory cells into the CNS [1]. ART may also contribute to stroke indirectly by increasing life expectancy and thus increasing the incidence of traditional vascular risk factors [2]. Though ART is the mainstay of HIV treatment, the implications for cerebrovascular disease with certain therapies should be considered in ART selection.

Given the known increased incidence of stroke in the HIV population, it is vital that clinicians consider more than TOAST criteria [12] when determining stroke etiology. This requires more nuanced testing to determine HIV status and control, the presence of opportunistic infections, HIV vasculopathy or a concurrent coagulopathy. This work up in turn determines appropriate secondary prevention, which often varies in this patient population. The management algorithm as presented provides a broad framework for clinicians when evaluating patients with HIV in the setting of acute stroke.

## Figures and Tables

**Figure 1:**
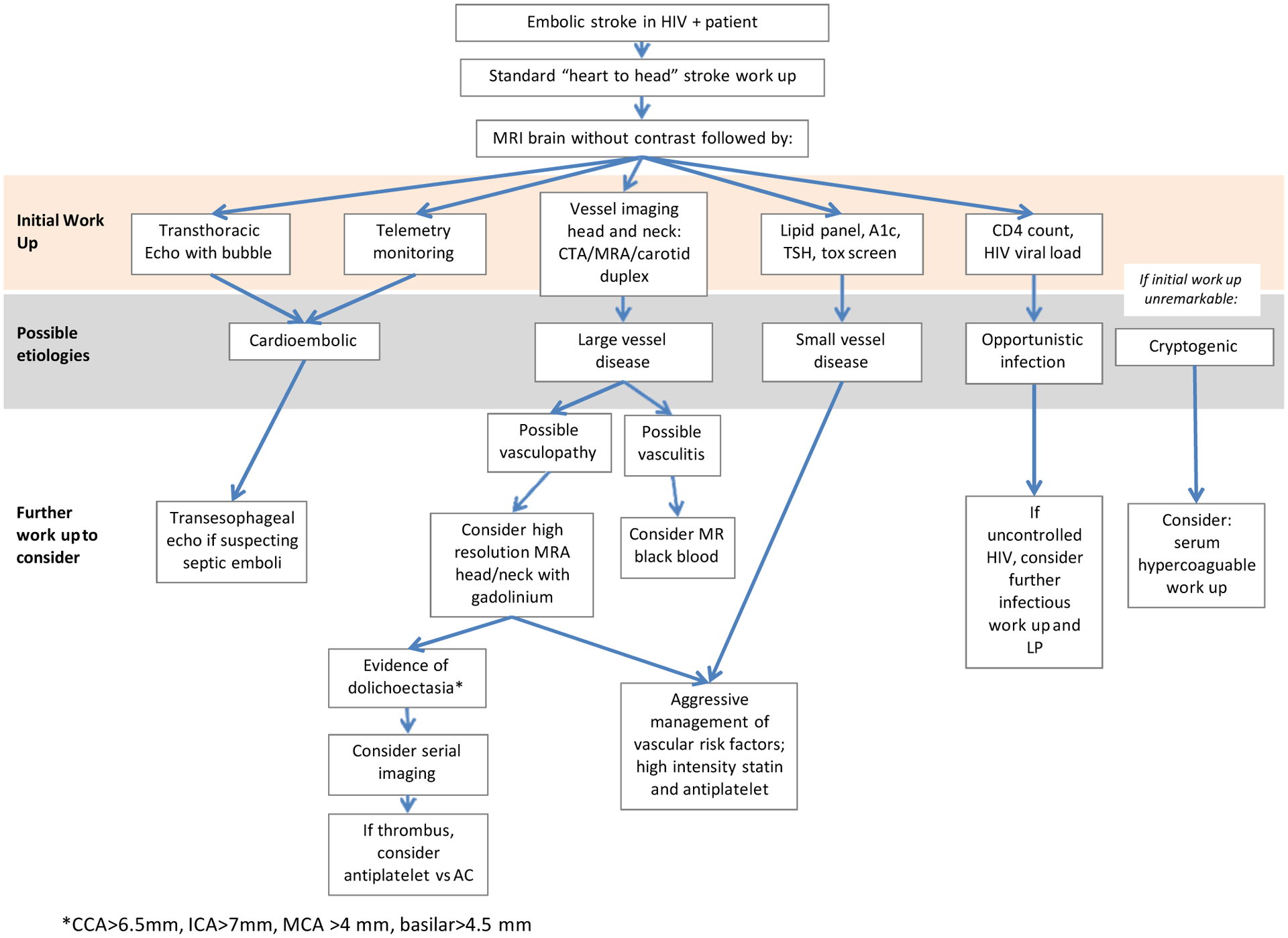
Proposed algorithm for inpatient management of ischemic stroke in HIV.
